# Editorial board

**DOI:** 10.1080/0886022X.2025.2480329

**Published:** 2025-04-07

**Editor-in-chief**

**Tibor Fülöp, MD, PhD** (Professor) – Medical University of South Carolina, Charleston, South Carolina, USA

**Deputy editors**

**Mihály Tapolyai, MD, PhD** (Nephrologist) – St. Margit Hospital, Budapest, Hungary and Ralph H. Johnson Veterans Affairs Medical Center, Charleston, South Carolina, USA

**Wisit Cheungpasitporn, MD** (Professor) – Mayo Clinic, Rochester, Minnesota, USA

**Social media editor**

**Wisit Cheungpasitporn, MD** (Nephrologist) – Mayo Clinic, Rochester, Minnesota, USA

**Senior Associate editors**

**Monika Gööz, MD, PhD** (Associate Professor) – Medical University of South Carolina, Charleston, South Carolina, USA (Basic Sciences)

**Fan He, MD, PhD** (Professor) – Tongji Medical College, Huazhong University of Science and Technology, Wuhan City, Hubei Province, China (Clinical Sciences)

**Wisit Kaewput, MD** (Associate Professor, Nephrologist, Lecturer and Deputy Head of Department) – Phramongkutklao Hospital and Phramongkutklao College of Medicine, Bangkok, Thailand (Clinical Epidemiology, Meta-Analyses and Systemic Reviews)

**Csaba Kopitko, MD, PhD** (Nephrologist) – Uzsok Teaching Hospital, Semmelweis University, Budapest, Hungary (Critical Care and Perioperative Sciences)

**Guisen Li, MD, PhD** (Chief Physician) – Sichuan Academy of Medical Sciences and Sichuan People’s Hospital, Chengdu, Sichuan, China (Basic Sciences)

**Karim M. Soliman, MD, PhD** (Associate Professor) – Medical University of South Carolina, Charleston, South Carolina, USA (Transplantation Sciences)

**Charat Thongprayoon, MD** (Assistant Professor and Senior Associate Consultant) – Mayo Clinic, Rochester, Minnesota, USA

**Ningning Wang, MD, PhD** (Professor) – The First Affiliated Hospital of Nanjing Medical University, Nanjing, China

**Guangtao Xu, MD** (Researcher) – Jiaxing University, Jiaxing, Zhejiang, China (Clinical Sciences)

**Zhongheng Zhang, MD** (Nephrologist) – Sir Run Run Shaw Hospital, Zhejiang University School of Medicine, Hangzhou, China

**Associate editors**

**Sohail Abdul Salim, MD** (Adjunct Assistant Professor and Nephrologist) – University of Mississippi Medical Center, Jackson, Mississippi, USA and Southwest Kidney Institute, Phoenix, Arizona, USA (Clinical Sciences)

**Jianbo Qing, MD** (Doctor of Medicine) – Zhejiang University, Hangzhou, China

**Ken Sakai, MD, PhD** (Professor) – Toho University Faculty Medicine, Tokyo, Japan

**J. Pedro Teixeira, MD** (Assistant Professor) – University of New Mexico, Albuquerque, New Mexico, USA

**Michael E. Ullian, MD** (Professor) – Medical University of South Carolina, Charleston, South Carolina, USA (Clinical Sciences) (advisory capacity)

**Biostatistics and statistical consultant advisor**

**Zsolt Lengvárszky, PhD** (Department Chair) – Louisiana State University Shreveport, Shreveport, Louisiana, USA

Section editors*

***Acute kidney injury***

**Jonathan Samuel Chávez Iñiguez, MD** (Chief of Nephrology) – Universidad de Guadalajara, Guadalajara, Mexico

**Hongqi Ren, MD, PhD** (Director) – Nanjing University School of Medicine, Nanjing, China

**Bijin Thajudeen, MD** (Associate Professor) – Banner University of Arizona, Tucson, Arizona, USA

***Anemia and hematologic disorders***

**A. Emre Eşkazan, MD** (Professor) – Istanbul University-Cerrahpaşa, Istanbul, Turkey

***Artifical intelligence and machine learning***

**Hatem Ali, MBBCh, MSc** (Clinical Research Fellow) – University Hospitals of Coventry and Warwickshire, Coventry, UK

**Zhongheng Zhang, MD** (Nephrologist) – Sir Run Run Shaw Hospital, Zhejiang University School of Medicine, Hangzhou, China

***Basic sciences investigations***

**Ahmed I. Adelkader, MD, PhD** (Transplant Nephrology Fellow) – Medical University of South Carolina, Charleston, South Carolina, USA and Mansoura University, Mansoura, Egypt

**Alejandro R. Chade, MD** (Professor and NextGen Precision Health Investigator) – University of Missouri-Columbia, Columbia, Missouri, USA

**Natalia Stepanova, MD, PhD, DSc.Med** (Professor) – Institute of Nephrology of the National Academy of Medical Sciences of Ukraine, Kyiv, Ukraine

***Bone-mineral and electrolyte disorders***

**Chen Fu, MD** (Nephrologist) – China Beijing Jishuitan Hospital Capital Medical University, Beijing, China

**Gheun-Ho Kim, MD, PhD** (Professor) – Hanyang University College of Medicine, Seoul, Korea

**Ningning Wang, MD, PhD** (Professor) – The First Affiliated Hospital of Nanjing Medical University, Nanjing, China

***Cardio-renal physiology and disease processes***

**Alejandro R. Chade, MD** (Professor and NextGen Precision Health Investigator) – University of Missouri-Columbia, Columbia, Missouri, USA

***Chronic kidney Disease and progression***

**Sebastjan Bevc, MD, PhD** (Professor) – University Medical Center Maribor and University of Maribor, Maribor, Slovenia

**Xuxia Gao, MD, PhD** (Nephrologist) – Capital Medical University Affiliated Beijing Anzhen Hospital, Beijing, China

**Liliana Gadola, MD** (Adjunct Professor) – Universidad de la Republica Facultad de Medicina, Montevideo, Uruguay

**Fan Yi, PhD** (Professor) – Shandong University, Jinan, Shandong, China

***Critical Care nephrology and continuous kidney replacement therapy***

**Jorge Castaneda, MD** (Assistant Professor) – University of Mississippi Medical Center, Jackson, Mississippi, USA

**Abduzhappar Gaipov, MD, PhD** (Associate Professor) – Nazarbayev University School of Medicine, Nur-Sultan City, Kazakhstan

**J. Pedro Teixeira, MD** (Assistant Professor) – University of New Mexico, Albuquerque, New Mexico, USA

***Glomerulonephritis and immunologic disorders***

**Andrea Angioi, MD** (Nephrologist) – ‘Giuseppe Brotzu’ Hospital, Cagliari, Italy

**Ana de Lurdes Agostinho Cabrita Vieira, MD** (Nephrologist) – University Hospital Center of Algarve, Faro, Portugal

**Stefan Gabriel MD, PhD** (Nephrologist) – Carol Davila University of Medicine and Pharmacy, Bucharest, Romania

**Nicolas Hanset, MD** (Nephrologist) – University Hospital Saint-Luc, Brussels, Belgium

***Hemodialysis and peritoneal dialysis***

**Gaetano Alfano, MD** (Nephrology Medical Director) – University Hospital of Modena, Modena, Italy

**Jin-Bor Chen, MD** (Nephrologist) – Kaohsiung Chang Gung Memorial Hospital, Chang Gung University College of Medicine, Kaohsiung, Taiwan

**Surapon Nochaiwong, PharmD** (Lecturer) – Chiang Mai University, Chiang Mai, Thailand

***Hypertension and volume management***

**Ana de Lurdes Agostinho Cabrita Vieira, MD** (Nephrologist) – University Hospital Center of Algarve, Faro, Portugal

**S. Mehrdad Hamrahian, MD** (Nephrologist) – Thomas Jefferson University, Philadelphia, Pennsylvania, USA

**Gheun-Ho Kim, MD, PhD** (Professor) – Hanyang University College of Medicine, Seoul, Korea

***Imaging technologies in nephrology***

**Alejandro R. Chade, MD** (Professor and NextGen Precision Health Investigator) – University of Missouri-Columbia, Columbia, Missouri, USA

***Medical education and training***

**Yavuz Ayar, MD** (Associate Professor) – University of Health Sciences, Bursa Faculty of Medicine, Bursa, Turkey

**Liliana Gadola, MD** (Adjunct Professor) – Universidad de la Republica Facultad de Medicina, Montevideo, Uruguay

***Nephrolithiasis and urolithiasis***

**Natalia Stepanova, MD, PhD, DSc.Med** (Professor) – Institute of Nephrology of the National Academy of Medical Sciences of Ukraine, Kyiv, Ukraine

***Nephrology nursing and the patients’ voice***

**Amy Perry, MSN, APRN, BC, CNN-NP 9** (Nurse Practitioner) – Ralph H. Johnson Veterans Affairs Medical Center, Charleston, South Carolina, USA

***Pediatric nephrology***

**Sihem Darouich, MD, MSc** (Professor) – University of Tunis El Manar, Tunis, Tunisia

***Pharmacology and nephro-oncology***

**Jorge Castaneda, MD** (Assistant Professor) – University of Mississippi Medical Center, Jackson, Mississippi, USA

**Christian A. Koch, MD, PhD, Medhabil, MACE** (Professor and Chief) – University of Florida, Jacksonville, Florida, USA

**Jolanta Malyszko, MD, PhD** (Professor) – Medical University of Warsaw, Warsaw, Poland

***Transplantation***

**Orsolya Cseprekál, MD, PhD** (Senior Lecturer) – Semmelweis University, Budapest, Hungary

**Miklos Z. Molnar, MD, PhD** (Professor and Medical Director) – University of Utah, Salt Lake City, Utah, USA

**Ken Sakai, MD, PhD** (Professor) – Toho University Faculty Medicine, Tokyo, Japan

* The Section Editors act as Topic Advisors to the editor and associate editor team and do not handle papers directly unless they are also listed as assocate editors.

**Editorial board members**

**Moh’d Mohanad A. Al-Dabet, PhD** (Associate Professor) – American University of Madaba, Amman, Jordan

**Abdullah K. Al-Hwiesh, MD** (Professor and Consultant) – Imam Abdulrahman Bin Faisal University, Al-Khobar, Saudi Arabia

**Petar Avramoski, MD, PhD** (Professor) – St. Clement of Ohrid University, Bitola; Clinical Hospital – Bitola, North Macedonia

**Mickaël Bobot, MD, PhD** (Nephrologist) – Aix Marseille Université, Marseille, France

**Tiffany N. Caza, MD, PhD** (Nephropathologist and Scientist) – Arkana Laboratories, Little Rock, Arkansas, USA

**Ahmed Daoud, MD, PHD** (Lecturer) – Cairo University, Cairo, Egypt

**Shao-bin Duan, MD, PhD** (Chief Physician and Professor) – The Second Xiangya Hospital of Central South University, Changsha, China (Basic Sciences)

**Youssef Farag, MD, PhD, MPH** (Faculty Director, Associate Faculty and Senior Medical Director) – Harvard Medical School, Boston, Massachusetts, USA

**Wayne Fitzgibbon, PhD** (Associate Professor) – Medical University of South Carolina, Charleston, South Carolina, USA

**Winston Wing-Shing Fung, MBBChir** (Associate Consultant and Honorary Assistant Professor) – Chinese University of Hong Kong, Hong King, China

**Mohammed Abdel Gawad, MD, ESENeph** – Newgiza University, Giza, Egypt

**Quan Hong, PhD** (Nephrologist) – Chinese PLA General Hospital, Beijing, China

**Yuh-Shan Ho, PhD** (Director and Associate Professor) – CT HO Trend, Tapei, Taiwan

**Ivica Horvatic, MD, PhD** (Nephrologist) – Dubrava University Hospital, University of Zagreb, School of Medicine, Zagreb, Croatia

**Bo Hu, MD** (Associate Professor) – Jiaxing Hospital of Traditional Chinese Medicine, Jiaxing University, Zhejiang, China

**Jun Jiang, MD** (Nephrologist) – The First Affiliated Hospital of USTC, University of Science and Technology of China, Hefei, China

**Priyanka Khandelwal MD, DM** (Nephrologist) – All India Institute of Medical Sciences (AIIMS), New Delhi, India

**Yafeng Li, MD, PhD** (Professor and Director) – Shanxi Medical University, Taiyuan, China

**Tongqiang Liu, PhD** (Nephrologist) – Nanjing Medical University, Changzhou, Jiangsu, China

**Alisson D. Machado, PhD** (Professor) – University of Sao Paulo, Sao Paulo, Brazil

**Shan Mou, MD, PhD** (Professor and Director Physician) – Shanghai Jiao Tong University, Shanghai, China

**Lovelesh Nigam, MD** (Associate Professor) – Institute Of Kidney Disease And Research Centre, Dr. H.L. Trivedi Institute of Transplantation Sciences (IKDRC-ITS), Asarwa, Ahmedabad, India

**Yannick Nlandu Mayamba, MD** (Nephrologist) – University of Kinshasa, Kinshasa, Democratic Republic of the Congo

**Ákos G. Pethő, MD, PhD** (Assistant Professor) – Semmelweis University, Budapest, Hungary

**László Rosiváll, MD, PhD, DSc Med, Medhabil** (Professor) – Semmelweis University, Budapest, Hungary

**Ernesto Sabath, MD** (Assistant Professor) – Hospital Ángeles Querétaro, Centro Sur Queretaro, Queretaro, Mexico

**Satoshi Shimada, MD, PhD** (Instructor) – Medical College of Wisconsin, Milwaukee, Wisconsin, USA

**László Szabó, MD** (Consultant Transplant Surgeon) – University Hospital of Wales, Cardiff, UK

**Rui-Zhi Tan, MD** (Associate Professor) – Southwest Medical University, Luzhou, China

**Kiyotaka Uchiyama, MD, PhD** (Associate Professor) – International University of Health and Welfare, Chiba, Japan

**Yonggui Wu, MD** (Professor) – Anhui Medical University, Hefei, China

**Wan Xin, MD** (Nephrologist) – Nanjing Medical University, Nanjing, China

**Zailing Xing, MS, PhD(c)** (Phd Candidate) – University of South Florida, Tampa, Florida, USA

**Takahiro Yajima, MD** (Research Director) – Matsunami General Hospital, Gifu, Japan

**Jianyun Yan, PhD** (Professor) – Zhujiang Hospital of Southern Medical University, Guangzhou, China

**Hongliang Zhang, MD, PhD** (Director) – National Natural Science Foundation of China, Beijing, China

**Previous editor**

**William F. Finn, MD** – University of North Carolina at Chapel Hill, North Carolina, USA

